# Trauma-informed healthcare systems: an evaluation of trauma-informed care training for hospital-based healthcare professionals in the aftermath of the 2023 earthquakes in Türkiye

**DOI:** 10.1093/heapol/czae118

**Published:** 2024-12-10

**Authors:** Zeynep Şimşek, Büşra Uğur

**Affiliations:** Faculty of Health Sciences, Trauma and Disaster Studies Applied Mental Health Program, Istanbul Bilgi University, Hacıahmet, Kurtuluş Deresi Cd. No: 19, Beyoğlu/İstanbul 34440, Turkey; Faculty of Health Sciences, Trauma and Disaster Studies Applied Mental Health Program, Hacıahmet, Kurtuluş Deresi Cd. No: 19, Beyoğlu, Istanbul 34440, Turkey

**Keywords:** trauma-informed care, training, earthquake, health systems, Türkiye, healthcare workers’ training intervention, postdisaster responses, occupational health and safety

## Abstract

Disasters are complex global problems with an increasing impact with rising prevalence of associated illness, mortality, and intensifying health inequities. In recent years, there has been an emphasis on integrating trauma-informed care approaches into health policies and protocols. The purpose of the current study was to investigate the benefits of a trauma-informed healthcare training program for hospital-based healthcare providers with a focus on knowledge acquisition, empowerment of professional practice, and personal well-being. The program was implemented in the aftermath of the 2023 earthquakes in southeastern Türkiye. The training consisted of four modules, developed based on psychological trauma theories and behavior change theories, and was evaluated using a mixed-methods approach. Assessments were conducted at the end of the training program, at baseline, and at a 6-month follow-up. A structured questionnaire including items covering the content of the training, trainer effectiveness, and program suitability was administered at the end of the training program. At 6 months, participants completed an 18-item follow-up questionnaire which assessed their understanding of the principles of the trauma-informed care approach. The Maslach Burnout Inventory (MTI) was also administered, and themes regarding the impact of the training program were extracted through in-depth individual qualitative interviews. Data were obtained from 501 program participants. The intervention program was found to improve healthcare workers’ understanding of trauma, professional practices, and interpersonal relationships, and significantly reduced symptoms of burnout. These results demonstrate the critical role of trauma-informed training programs in hospitals in disaster-affected regions, especially when assistance to survivors will be enhanced by strengthening healthcare workers’ resilience and improving their perceptions of service efficacy and value. The study highlights the need for more widespread adoption of these training initiatives and emphasizes that they may play significant future roles in transforming trauma-informed healthcare systems in disaster-prone countries and regions.

Key messages

- Disasters are becoming more frequent and severe, contributing to escalating rates of illness, mortality, and widening health disparities globally.
- A trauma-informed healthcare training program was developed and implemented for hospital-based healthcare providers in the aftermath of the 2023 earthquakes in Türkiye, with an emphasis on enhancing knowledge, professional practice, and personal well-being.
- The intervention demonstrated significant improvements in healthcare workers’ understanding of trauma, professional practice, interpersonal dynamics, and a marked reduction in stress symptoms over time.
- The findings underscore the necessity for broader integration of trauma-informed care training in healthcare systems, particularly in disaster-prone areas, to foster resilience and optimize care delivery in response to complex traumatic events.

## Introduction

According to the World Health Organization (WHO, 1992), disasters are tragic occurrences that cause deaths and financial and environmental losses and disrupt social functioning, exceeding the capacity of the affected communities to cope with available resources for them (United Nations (UN) 1992). Since 1900, there have been more than 26 000 disaster records that met at least one of the criteria of causing at least 10 deaths (including dead and missing) and 100 affected people (including injured and homeless) based on the International Disaster Database (EM-DAT) criteria requiring a declaration of a state of emergency and call for international assistance (CRED, 2023). Türkiye is located on the highly seismically active Anatolian Plate, on which massive earthquakes occurred throughout history. Since the year 1900, 20 earthquakes with a magnitude of over 7 have occurred in these lands, placing Türkiye among the top-listed countries affected by earthquakes. Türkiye has experienced 269 earthquakes that caused loss of lives and economic damage between 1900 and 2023. Of these, the top three earthquakes that claimed the most lives and inflicted the most severe damage were the 2023 Kahramanmaraş Earthquake. On 6 February 2023, moment magnitude (Mw) 7.8 earthquake struck the Kahramanmaraş province in southeastern Turkey near Pazarcık, followed by a doublet 7.5-Mw earthquake hours later in the Elbistan region of the same province, with numerous aftershocks, resulting in over 50 000 deaths, and economic cost over 104 billion US dollars (Walika et al. 2023, Kahramanmaraş ve Hatay Depremleri Raporu, 2023).

Beyond the mounting global toll of death, injury, and material damage, the seriousness of ensuing traumatic stress due to loss of trust and sense of control represents major challenges at times of disasters. According to The Diagnostic and Statistical Manual of Mental Illnesses (DSM-5) fifth edition (DSM-5) (APA, 2013), trauma is a condition experienced by an individual through direct experience, witnessing in person events as it occurred to others, and learning about a traumatic event related to close family members or friends or due to professional exposure to the disturbing details of a traumatic event, involving actual or threatened death, serious injury, or sexual violence. A study by the WHO in 24 countries found that 70.4% of participants had been exposed to trauma (Kessler et al. 2017). A meta-analysis revealed that individuals with severe mental illness are more likely to have experienced interpersonal trauma or traumatic events compared to the general population (Mauritz et al. 2013).

Although there remains a lack of universal consensus on trauma-informed care, four key guidelines of trauma care (the 4R) have been provided by the Substance Abuse and Mental Health Services Administration Concept of Trauma and Guidance for a Trauma-Informed Approach (SAMHSA, 2014) relevant to health systems: (i) recognition of widespread impact of trauma “Recognize”, (ii) identification of symptoms of trauma among clients and staff “Realize”, (iii) integration of trauma-informed care in policies and practices “Respond”, and (iv) prevention of retraumatization “Resist”. Such a trauma-informed approach is not just a specific service model or set of rules to follow but a comprehensive organizational and institutional process aimed at fostering environments and relationships necessary for healing and preventing of retraumatization (Sweeney et al. 2018). In behavioral health settings, trauma-informed care has initially focused on childhood adversities due to their lifelong impact on future emergence of cancer, cardiovascular disease, depression, substance use disorder, and suicide. However, the increase in disasters worldwide necessitates consideration of trauma-informed approaches across all traumatic experiences not just in the context of childhood (Sowder et al. 2018, Chang et al. 2019). The growing interest in trauma-informed care within the health system therefore aims to prevent retraumatization, accelerate healing, and reduce healthcare costs, especially among healthcare professionals with traumatic experiences (D’Andrea et al. 2013, Kessler et al. 2014, Raja et al. 2015, Bowen and Murshid 2016). Furthermore, studies show that patients themselves receiving healthcare services face an increased risk of retraumatization throughout the process, from their initial interaction with healthcare providers to the interventions and settings they encounter (Coles and Jones 2009, Middleton et al. 2019, Goddard 2021).

Trauma-informed care as an approach to health and humanitarian services therefore entails consideration of a more holistic history of trauma, recognizing its widespread impact, shifting from “What’s wrong with you?” to “What happened to you?,” understanding how the traumatic event affects encountered individuals’ lives, and focusing and promoting their healing (Felitti et al. 1998, Coles and Jones 2009, SAMHSA, 2014, Goldstein et al. 2018, Beattie et al. 2019). Studies demonstrate that trauma-informed care in the health system increases patients’ adherence to treatment, accelerates healing, enhances the well-being of healthcare workers, increases their confidence and awareness, changes their attitudes toward patients, improves trust-based communication, and reduces mental issues like anxiety, secondary traumatization, post-traumatic stress disorder, depression, dissociation, postpartum depression, and psychological dysregulation (Helitzer et al. 2011, Ford and Blaustein 2013, Hodgdon et al. 2013, Green et al. 2016, Palfrey et al. 2019, Williams et al. 2023, Chu et al. 2024). Despite the growing recognition of the importance of trauma-informed approaches, many healthcare workers have not received specific training in this arena, either in their practices or during the employment process (Piper et al. 2021, Roberts and Murray 2023).

As in many countries, trauma-related stress-related care in Türkiye is not sufficiently emphasized in most undergraduate programs of health professions (Courtois and Gold 2009). Currently, there is one program at the authors’ institute for the dissemination of trauma-informed care. Academicians of this program have incorporated lessons focusing on traumatic stress and approaches into in-service training programs for primary healthcare and social services, especially those serving immigrants, under both the Ministry of Health and the Ministry of Family and Social Services. These training programs aim to inform workers about the effects of psychological trauma and the principles of trauma-informed practice, fostering attitudes and behaviors that support healing. The current literature suggests that these training programs, even though typically lasting a day, have shown effectiveness in practice through pre- and post-tests; nevertheless, further research is needed to evaluate their long-term impact (Wilson et al. 2017; Palfrey et al. 2019).

As noted earlier, given its location on the highly seismic risk-prone Anatolian Plate, with experience of 90 major earthquakes between 1900 and 2022 meeting the EM-DAT disaster criteria, with at least one magnitude 5.0 or higher each year (Walika et al. 2023), and given the experience of the 1999 Marmara earthquakes, an in-service “Disasters and Psychological Trauma” training was planned in the 2022–2023 University and İstanbul Health Directorate Applied Training Protocol to start in February 2023, in preparation for anticipated future earthquakes in the Istanbul province. The emergence of the February 6, Kahramanmaraş earthquake, and the ensuing transfer of several patients and injured to various provinces, including Istanbul, and the mobilization of healthcare workers across provinces to and from the earthquake area was therefore a serendipitous occurrence. The “Disasters and Psychological Trauma” training, planned before the earthquake, was therefore conducted in March 2023 targeting the healthcare workers located in major hospitals in Istanbul engaged in the care of the earthquake victims, under the title “Trauma-Informed Care in Disasters.” The objective of the study was to assess the efficacy of implementing a comprehensive day-long training program designed to address the aftermath of disasters, specifically focusing on earthquakes.

## Methods

### Study design

This study aimed to determine the influences of 1-day trauma-informed care training given to health workers employed in 11 different city hospitals and whether the training affected the occupational health, professional practices, and relationships within their work and social environments. A healthcare worker was eligible to participate in the intervention if they were employed in direct care and included the following categories of healthcare employees: doctors, nurses, social workers, psychologist, and health technicians. The qualitative and quantitative assessments were conducted at the end of the training and at the 6-month follow-up. The objective was to reach 20% of health workers at the 6-month follow-up depending on time and budget restrictions; however, the actual result was approximately 17% (with a response rate of 90%). The assessments were conducted at the end of the training and at the 6-month follow-up. The study was approved by the Istanbul Health Directorate and the hospital administration. The interviews with the participants were conducted based on confidentiality, the term “K” was used to represent the health workers without including names, and the numbers “K1, K2, K3, K4” were given next to each statement (K1: obstetric nurse; woman; K2: radiology technician, male; K3: psychiatric nurse, female; K4: nurse, female). IBM SPSS 26.0 statistical program was used for data entry and analysis. The analyses employed descriptive statistics (mean, standard deviation, and percentage), Pearson correlation, *t*-test, and chi-square analysis.

### Participants

A total of 501 healthcare workers employed in hospitals participated in the trauma-informed care training. A total of 69.5% of the healthcare workers were women, and 30.5% were men. The mean age of the participants was 36.2 ∓  9.7 years (23–58). When the distribution according to professions was analyzed, 31.5% were nurses; 12.4% were physicians; 38.1% were psychologists, social workers, and child development specialists; and ∼24.7% were health technicians. At the 6-month interval, the online questionnaire was completed by 89 health workers out of 100 participants, selected via systematic sampling from a list of 501 participants. No statistically significant differences were found according to gender (65.2% were females and 34.8% were males), age (35.1 ± 8.9), or occupation (*P* > .05).

### Training module development and administration

The training module was prepared based on the neurobiological and psychosocial theories of mental trauma, principles of trauma-informed care (safety, trustworthiness and transparency, peer support, collaboration and reciprocity, empowerment, choice and participation, and cultural and gender sensitivity), components of trauma-informed care (understanding the prevalence and manifestations of psychological trauma, appropriate approach to stress reactions, preventing retraumatization, and protecting and maintaining worker well-being), theories of behavior change (health belief model and reasoned action approach), and real case environments from the field experiences of experts. The training program consisted of four sessions administered between 09:30 a.m. and 05:00 p.m, the contents of which are described further.

#### Session one—“understanding disaster risk”

The epidemiology of global and national disasters caused by natural events was explained with the aim of increasing perceptions of seriousness and high likelihood of lifelong encountering of such disasters by health professionals emphasizing the need to gain knowledge and transform behavior preparedness.

#### Session two—“basic principles of trauma-informed care”

The common cognitive, behavioral, physical, and social reactions to mental trauma in individuals and societies due to neurobiological and psychosocial mechanisms were explained with case examples, emphasizing basic principles. A central principle was the “healthy life plan in disasters” which included an explanation of physical, emotional, social, intellectual, and spiritual life domains to prevent secondary traumatization and burnout among health workers and to develop resilience based on occupational health and safety. Following this behavioral principle, trust-based relationships with disaster survivors, rebuilding and strengthening bonds, respect for human dignity and rights, prevention of discrimination, prioritizing at-risk groups, promoting interdisciplinary shared responsibility, and ensuring active participation of the community to regain the lost sense of trust and control were highlighted. The importance of creating a safe physical and emotional environment (e.g. preventing infectious diseases, reducing the risk of chemical exposure, exercises to develop a problem-solving approach, keeping complete and accurate records for continuity, and being accessible) was explained with examples. Steps to increase hope, recovery, and traumatic growth by providing knowledge and skills to control and eliminate hazards that increase the risk of developing physical and mental disorders (such as using chlorine tablets, wearing masks, and employing a problem-solving approach) were also explained.

#### Session three—“understanding and approach to traumatic stress reactions”

The session explained the importance of preventing mental disorders, in terms of early diagnosis, early treatment, relapse prevention, promotion of resilience, understanding, and positive reactions after disasters. It also emphasized that gaining healthy life skills through the prevention of retraumatization, facilitating return to routine practices by opening space for grief and sorrow, and the effect of facilitating return to normative relationships on regaining a sense of trust and control were explained with case examples.

#### Session four—“triage, psychological first aid, and psychoeducation”

Due to the proximity and the high impact of the earthquake in the region, triage, an eight-step psychological first aid protocol, integrated with physical first aid, and psychoeducation practices were included.

### Evaluating the impact of training intervention

The impact of 1-day trauma-informed care training was assessed using a mixed-methods approach, encompassing both quantitative and qualitative techniques, at both baseline (Month 0) and the 6-month follow-up (Month 6).

A baseline evaluation was conducted following the training program. Participants were asked to respond to 11 statements on a Likert scale (1–5) to assess the training’s comprehensibility, usefulness, and process. Additionally, participants were requested to score (1–5) the new information they had gained from the training and were posed an open-ended question: “Please describe in a few sentences how you felt today.”

At the 6-month follow-up, both quantitative and qualitative data were collected. In the quantitative study, the MTI and the Trauma-Informed Care Assessment Form were administered to the participants via Google Forms. The qualitative data were collected through in-depth interviews with four healthcare professionals using a semistructured questionnaire. The interviews lasted for, on average, 35 min.

Thematic analysis with a six-phase approach, as detailed by Braun and Clarke (2012), was applied to the answers to the open-ended question in the baseline assessment and in-depth interviews conducted at 6 months. This approach was used to systematically identify and organize patterns of meaning in the data to generate a shared understanding of the experiences relevant to the training. The researchers engaged in a dialogic process while coding in order to gain a deeper comprehension of the data and to facilitate its analysis. Once all the in-depth interviews had been completed and transcribed, the researchers familiarized themselves with the data, discussed any patterns that emerged in the transcripts, and generated initial codes. Through a process of shared dialogue, the researchers identified the need to recode and collapse the codes into broader themes. As the themes and subthemes were defined, the researchers continued reiterating the process of returning to the data to gather quotes and examples, resulting in three overarching themes.

## Trauma-informed care assessment form

This form consisted of 18 items on a 5-point Likert scale developed by researchers. The items included recognizing the widespread impact of trauma, recognizing the symptoms of mental trauma in service providers and recipients, preparing a healthy life plan, preventing retraumatization, and the impact of trauma-informed care on work, friend, and family relationships. The internal consistency of the form is 0.97.

## MTI

The MTI developed by Maslach and Jackson (1981), the validity and reliability study conducted by Ergin in Turkey (Ergin 1992), consists of 22 questions on a 5-point Likert scale. It has three dimensions: emotional exhaustion (EE), personal accomplishment (PA), and depersonalization (DE) (7). EE and DE dimensions include negative responses, whereas the PA dimension includes positive responses. Scores are calculated separately for each scale. Since there is no cutoff value for the scores obtained from the subscales, no distinction can be made as to whether there is burnout. Individuals experiencing burnout are expected to have high EE and DE scores and low PA scores. Statements numbered 1, 2, 3, 6, 8, 13, 14, 16, and 20 are attitude statements used to measure EE; statements numbered 5, 10, 11, 15, and 22 are used to measure DE; statements numbered 4, 7, 9, 12, 17, 18, 19, and 21 are used to measure PA. According to the reliability analysis results, Cronbach’s alpha coefficients were 0.89 for the EE subdimension, 0.77 for the DE subdimension, and 0.74 for the personal failure subdimension. According to the reliability analysis results, Cronbach’s alpha coefficients are 0.89 for the EE subdimension, 0.77 for the DE subdimension, and 0.74 for the personal failure subdimension.

## Results

### Evaluation of the training content and process

Table 1 shows the means and standard deviations of the scores given between 1 and 5 for the items related to the content and process of the training. When the mean scores of the items are compared, the mean scores obtained from the items of the trainer’s knowledge of the subject and willingness, giving appropriate feedback, the appropriateness of the content of the training, and the training increasing motivation are significantly higher than the other items (*P* < .05). In addition, the mean score obtained from “new information they acquired in the training” is 4.09 ∓ 0.90, and the mean score obtained from how the information acquired in the training made them feel is 4.30 ∓ 0.91.

**Table 1.** The results of the evaluation given to the training’s content and procedure items

Items about the training’s contents and procedure	*x̄*	Standard deviation
1. The aims and objectives of the training were clear and explicit	4.33	1.07
2. The scope of the training was up-to-date and adequate	4.25	1.15
3. The time allocated to the topic was sufficient?	4.05	1.39
4. The appropriate trainer was selected for the subject?	4.33	1.11
5. The training materials used were understandable and adequate	4.22	1.12
6. The content of the program was appropriate	4.47	0.96
7. The trainer was knowledgeable and enthusiastic	4.65	0.62
8. The trainer ensured the active participation of the participants	4.43	1.03
9. Participants were given constructive feedback	4.47	0.96
10. The training was motivational	4.47	1.01
11. The information I received contributed to my development.	4.35	1.17
12. Please rate the new knowledge you acquired today on a scale of 1 (very little new knowledge)-5 (a lot of new knowledge).	4.09	0.90
13. Please rate how the information you gained in this training made you feel on a scale of 1 (very bad) to 5 (very good).	4.30	0.91

The mean score obtained from the new information they acquired in the training is similar to the mean score obtained from the current adequacy of the scope of the training (*P* > .05). There was no significant relationship between the mean score obtained from each item and the duration of employment (*P* > .05).

Three themes were identified when the participants’ responses to the open-ended question “Please, write in a few sentences what you felt today?” were analyzed. When evaluated in general, the themes of occupational health and safety, which is at the center of the ethical principles of disasters; understanding and approaching stress reactions within the scope of a trauma-informed approach; and the impact of the educator with case examples emerged. Examples of each theme and written statements are given below. It is observed that the statements of the participants are consistent with the results given in Table 1.

### Qualitative results

Three themes were identified when the participants’ responses to the open-ended question “Please, write in a few sentences what you felt today?” were analyzed. When evaluated in general, the themes of occupational health and safety, which is at the center of the ethical principles of disasters; understanding and approaching stress reactions within the scope of a trauma-informed approach; and the impact of the educator with case examples emerged. Examples of each theme and written statements are given below. It is observed that the statements of the participants are consistent with the results given in Table 1.

#### Theme 1: Recognizing the importance of disaster, traumatic stress, and coping methods within the scope of occupational health and safety of health workers

The training allowed healthcare professionals to identify the prevalence of disasters and the elevated probability of such events occurring in their own lives. Furthermore, the training underscored the influence of disasters on emotional, cognitive, behavioral, and physiological processes, emphasizing the loss of trust and control experienced by those directly affected and those who assist. The majority of participants reflected on the broader context of their work, articulating a shift in perspective from a sense of absence to a sense of gratitude for their presence. “I am not working today, but now I am saying that I am glad I am here! Our country is a disaster country, and it is essential to be prepared.” This recognition of the necessity for preparedness further underscores the importance of integrating trauma-informed training programs into the health and safety framework.

Furthermore, participants emphasized the importance of addressing traumatic stress and developing coping strategies as integral components of occupational health and safety. For example, a participant wrote, “…Someone came and said that you are valuable too. Stop and breathe!” This statement underscores the significance of attending to workers’ physiological and psychological needs. Another participant wrote, “It was beneficial to participate in this training program to discuss strategies for reducing anxiety among health workers.” The training was a valuable opportunity for health workers to gain tools to manage anxiety effectively. For instance, a participant noted, “We did not consider stress to be an issue that could be addressed. It felt like part of our job. By the end of the training, I understood that we could not live with it.” This illustrates a transformative understanding that coping with stress is not merely an occupational hazard but an essential aspect of maintaining professional efficacy.

#### Theme 2: understanding and appreciating traumatic stress reactions

This training showed health workers the importance of creating a safe environment and bonding where emotions can be shared, while also ensuring physical safety. One participant noted “We came to recognize that the experience of a disaster constitutes a wholly distinct reality and that it is imperative to comprehend the stress reactions that accompany it before we can assist.” This statement underscores the importance of a foundational understanding of trauma reactions to facilitate adequate support. Another participant stated, “It is crucial to provide individuals affected by the disaster with the opportunity to express their emotions while also avoiding undue pressure.” This insight reflects an understanding of the trauma-informed care principle of “safety,” which is a core tenet of this approach.

The recognition of pain as an integral aspect of the human experience was a prominent subtheme. One participant stated, “Pain is an inherent and inevitable aspect of the human experience.” “This training was worthwhile even with the awareness that it would entail confronting painful experiences.” This perspective underscores the importance of acknowledging and validating emotional experiences as a crucial aspect of the healing process. One participant wrote a personal transformation through the training, stating, “I initially approached the training with the assumption that it was merely a learning experience. However, the insights I gained will significantly enhance my personal growth. Previously, I perceived the act of concealing one’s emotional distress as a form of ingenuity. However, after the training, I recognized that ingenuity primarily contributes to our overall well-being.” This reflection exemplifies the training’s profound impact on reshaping participants’ beliefs about emotional expression and its relation to overall well-being.

#### Theme 3: Characteristics of the trainer and the effect of case examples on learning

The efficacy of the training was found to be significantly influenced by the characteristics of the trainer and the incorporation of real-world case studies. One participant offered the following observation: “The training was useful. At times, the tone of voice made the topics approachable and relatable, while at other times, it delved into matters that touched upon my most sensitive spiritual concerns.” I experienced a sense of being understood while listening. I am grateful for the humanitarian presentation style. This underscores the significance of the trainer’s delivery style in fostering an empathetic and supportive learning environment.

Participants expressed appreciation for the practical relevance of case examples. One participant observed, “The examples were not solely from the book but also from the earthquake itself. This approach facilitated a deeper understanding beyond mere theoretical statements. While locating information in a book is relatively straightforward, it is not always easy to gain insights from first-hand experiences. This underscores the value of integrating experiential knowledge into training, enhancing the applicability of learned concepts to real-life situations.”

### Evaluation of the impact of the training at 6 months

Table 2 shows the means and standard deviations of the scores the participants gave to 18 items related to trauma-informed care in the sixth month of the training. When the averages of the scores given by the participants between 0 and 5 are examined, it is seen that they vary between 3.84 and 4.64. When the table is observed, it is seen that the participants learned the reactions that develop due to traumatic stress and approaches such as listening, empathic response, breathing exercises, and connecting with services appropriate to the need and understood the necessity of training all employees for trauma-informed service delivery. When the mean scores are analyzed, it is observed that the training empowered the employees by positively affecting their work success, friendships, and family relationships.

**Table 2.** Distribution of scores obtained from Trauma-Informed Care Assessment Form in the sixth month of training

Items	*x̄*	Standard deviation
I am aware of the reactions that occur after traumatic experiences.	4.40	0.77
What I have learned has helped me be patient.	4.22	0.90
I know what to do to return to a calm and balanced state when I get very angry while working.	4.06	0.92
I have become aware of my own emotions, behaviors, thoughts, and changes in my body.	4.10	0.97
I am aware that taking deep breaths and oxygenating with deep breaths activates the parasympathetic nervous system and calms me down.	4.34	0.81
I know that a healthy living plan strengthens the individual against traumatic experiences.	4.53	0.66
I am aware that helping someone grieving a loss is necessary.	4.44	0.74
I know that mourning after a traumatic experience accelerates healing.	4.39	0.81
I know that offering choices to the person I provide services to increases trust and a sense of control.	4.24	0.88
I know that supporting each other among colleagues helps to maintain the mental well-being of both service providers and recipients.	4.47	0.69
I know that listening helps normalize individual reactions.	4.36	0.77
I know that empathetic reactions help normalize stress responses.	4.37	0.73
Recognizing and establishing relationships with relevant individuals/institutions helps normalize stress responses.	4.46	0.62
I know the importance of all employees being trained in a trauma-informed approach for its integration into all services.	4.64	0.57
My performance in professional practices has improved.	4.01	1.0
Sharing what I have learned with other colleagues has empowered me.	3.98	1.0
It has positively contributed to my relationship with my colleagues.	3.87	1.18
It has positively affected my family relationships.	3.84	1.11

Table 3 shows the relationship between the total scores obtained from trauma-informed care items and the MTI subdimensions. As seen in the table, the total score of trauma-informed care/service delivery increases and the mean score of personal failure decreases. This finding is in parallel with the mean score obtained from the statement “My success in professional practices increased” given in Table 2.

**Table 3.** The relationship between the Trauma-Informed Care Assessment Form and the subdimensions’ total score of the MTI

MTI subdimensions	*x̄*	SS	Trauma-informed care total score	
			*R*	*P*
Desensitization	8.3	2.9	−0.085	0.431
EE	18.6	6.0	–0.461	0.661
PA	16.7	3.8	–0.453	0.001

### Qualitative results

Three overarching themes and subthemes resulted from the thematic analysis of the healthcare workers’ experiences, thoughts, and training application. This analysis is organized around three themes: (i) understanding and approaching traumatic stress reactions, (ii) realizing the importance of coping with stress within the scope of occupational health and safety, and (iii) training content and suitability of trainers. Overall, healthcare workers improved their understanding and utility of the trauma-informed care principles.

#### Understanding and approaching traumatic stress reactions

This theme encompasses the participants’ introspective accounts of their emotional and psychological responses to traumatic events, particularly in the context of the earthquake. Explanations for the three subthemes that emerged within the framework of this central theme are given below:

- Normalization of traumatic responses: A noteworthy finding is the participants’ acknowledgment of their emotional responses as typical reactions to traumatic experiences. For example, Participant 3 expressed, “In this training, I realized that these feelings are normal and represent a significant shift in my emotional processing.” This acknowledgment serves to mitigate feelings of isolation and distress, fostering a sense of solidarity among individuals who have experienced similar traumatic events.
- The emotional intensity experienced by the participants was considerable. The participants provided detailed accounts of the intense emotional distress they experienced during and in the aftermath of the earthquake. Participant 4 described feelings of detachment and breathlessness, which serve to illustrate the psychological disorientation that can accompany trauma. Such narratives underscore the critical necessity for the establishment of efficacious emotional support mechanisms within high-stress occupational settings, as they elucidate the profound impact of trauma on both mental health and occupational functioning.
- Cognitive reappraisal: The interviews revealed a significant cognitive shift among participants regarding their traumatic experiences. Participant 1 expressed, “Now I understand why this happened,” indicating a transition from confusion to cognitive clarity. This cognitive reappraisal is integral to the recovery process, as it enables individuals to contextualize their experiences, thereby facilitating the development of effective coping strategies.

#### Realizing the importance of coping with stress within the scope of occupational health and safety

This theme addresses the participants’ evolving understanding of self-care and its implications for their professional responsibilities and overall well-being.

- Self-care as a professional imperative: A critical insight from the interviews was the participants’ realization of the necessity of self-care within the context of their professional obligations. Participant 1 remarked, “We see it as unfair to care for ourselves when responding to disasters,” reflecting a prevalent belief that self-care is a form of selfishness. The training challenged this misconception, elucidating how the well-being of healthcare professionals directly influences the quality of patient care delivered.
- Emotional expression and processing: The significance of emotional expression emerged as a vital component of effective self-care. Participant 2 stated, “With the self-care sessions, I realized how important it is to express our emotions.” This recognition underscores the need for institutional frameworks that promote safe and supportive environments for emotional expression, which can significantly alleviate stress and enhance psychological well-being among healthcare workers.
- Empathy and emotional boundaries: Participants articulated the crucial balance between empathetic engagement and maintaining emotional boundaries. Participant 4 emphasized, “…it is essential to empathize with patients and maintain our emotional boundaries,” suggesting that such a balance is vital for sustaining emotional health while providing care. This insight points to the necessity of training programs that equip healthcare workers with skills to navigate the complexities of emotional engagement in high-stress situations.

## Training content and suitability of trainers

This theme reflects participants’ evaluations of the training program’s content and the effectiveness of its facilitators.

- Relevance and applicability of training content: Participants expressed appreciation for the practical relevance of the training, particularly the incorporation of real-life examples from the earthquake zone. Participant 1 noted, “…experts shared real examples to explain better the topics,” which enhanced relatability and underscored the training content’s applicability to their professional realities.
- Instructor quality and engagement: The participants highlighted the importance of instructor effectiveness in delivering impactful training. Participant 2 remarked on the strong oratory skills of one educator, stating, “…he explained every sentence as if he was living.” This connection between the instructor and participants fostered a more engaging learning environment, facilitating a more profound understanding and retention of the material.
- Case-based learning approaches: The training format utilized in case studies was particularly well-received by participants. Participant 4 stated, “I learned how to manage problems through cases,” indicating that this practical approach enhanced understanding and equipped participants with the necessary skills to apply their knowledge in real-world scenarios. Such an approach aligns with best practices in adult education, emphasizing experiential learning to foster competency and confidence.

## Discussion

The importance of trauma-informed healthcare systems is increasing due to the rising number of disasters and associated exposures worldwide. Trauma-informed care has three dimensions: capacity building of the workforce (training and awareness rising), trauma-focused services (rapid screening and evidence-based practices), and institutional practices (collaboration and coordination of services, trauma-informed policies, and procedures) (Hanson and Lang 2014). While training to build the capacity of staff continues to be provided, monitoring the impact of training programs and evidence-based development efforts has gained importance. This study aimed to evaluate the effect of 1-day trauma-informed care training given to healthcare professionals working in state hospitals in Istanbul province after the major February 6 earthquakes in southeastern Turkey. The training program included four sessions for health technicians, physicians, nurses, psychologists, social workers, psychologists, and all other healthcare workers together to understand the prevalence of disasters and complicated trauma, to recognize traumatic stress reactions based on cases, understand the relationship between receiving healthcare and retraumatization, and apply behavioral/ethical rules in disasters. Stress control methods, also referred to as self-care within the scope of occupational health and safety, triage, psychological first aid, and psychoeducation, were also included.

This study, in which the effect of the training was evaluated at the 6-month follow-up, revealed important results that may be useful in developing trauma-informed care training programs for healthcare professionals with a holistic approach. The content of the training and the fact that it was provided in a case-based interactive manner were evaluated as new and innovative and met the needs of health workers at all levels. The participants reported that the training they received improved their work, friend, and family relationships. In the prior literature, a study evaluating the effect of 1-day trauma-informed in-service training given to mental health workers after 3 months, like the findings of this study, reported that health workers acquired new knowledge and skills, continued to use the knowledge and skills they acquire in their health practices, and experienced a significant attitude change (Niimura et al. 2019). In a further study, Chukwu (2016) emphasized that the attributes such as facilitator disposition, the use of real-life examples, and active trainee participation significantly contribute to the effectiveness of training and that trainer characteristics are crucial determinants of training success and should be carefully considered in developing and implementing healthcare training programs (Shapiro et al. 2007, Chukwu 2016, Rawski et al. 2020, Aziz et al. 2022).

The literature also reports that trauma-informed training should cover all employees, including both management and support staff (SAMHSA, 2014). For this reason, in this study, the program was created with content that covers every employee working in the hospital regardless of job description, and case examples were given in a way to reveal the impact of the entire system. Research indicates that the principles of trauma-informed care can reduce burnout by fostering interdisciplinary collaborations among clinical staff, encouraging an egalitarian work environment rather than a hierarchical one, and promoting respect for each other’s professional boundaries and needs (Dawson-Rose et al. 2019, Roberts and Murray 2023). This study also found that the participation of all staff members in the health team in training and their learning about recognizing individual needs within the framework of cognitive empathy and establishing relationships with relevant people/institutions helped normalize stress responses. This increased solidarity and collaboration among them and the high average score obtained from the item “all staff should receive trauma-informed training” indicate the necessity of providing this training to all employees within the system, not just to clinical staff or specific professions. In this study, the 6-month evaluation showed that as knowledge of trauma-informed approaches increased, the score for the personal failure dimension of burnout significantly decreased, and a positive impact of the training on job success was reported.

A further significant finding of the study is that trauma-informed care enables workers to recognize the importance of addressing their health and needs, which is a fundamental principle in providing services to both providers and recipients during disasters. This awareness helps in understanding stress as a manageable risk factor. There is limited research in the literature demonstrating the impact of trauma-informed care practice on employees. In a study conducted by Hales et al. (2017), volunteers in the fields of mental health and substance use reported increased satisfaction with their relationships with their supervisors and their jobs, as well as with implementing institutional policies after 12 months of practicing trauma-informed care (Hales et al. 2017). This study also showed that employees require more trauma-informed self-care than institutional support to prevent secondary traumatization. Eastwood and Ecklund (2008) found in their study with 57 caregivers for residential children that those who perceived themselves as less competent in self-care and who socialized less with their families experienced higher levels of burnout.

Ensuring the physical and psychological health of employees and protecting them from workplace hazards are among the primary objectives of occupational health. It is known that workplace stress leads to mental, physiological, and neurobiological changes, causing cardiovascular and musculoskeletal diseases, among other occupational illnesses (LaMontagne et al. 2014). Neurobiological and psychosocial evidence shows that regular physical activity, dietary regulation, water consumption, adequate sleep, deep muscle relaxation, breathing exercises, meditation, strengthening social support systems, taking breaks, problem-solving skills, proper use of personal protective equipment, and time management protect against stress-related illnesses, increase productivity, and support overall well-being (van de Poll M et al. 2020, Qian et al. 2022). Therefore, all dimensions mentioned in the training have been addressed within the framework of preparing and implementing a healthy living plan, explained as a fundamental principle under occupational health and safety standards.

Effective interventions for job stress in healthcare workers in the literature include educational and work-hour reorganization, stress management programs, cognitive behavioral therapy and mindfulness-based psychotherapeutic interventions, and workplace counseling services (Firth-Cozens and Payne 1999; Tsarouchas et al. 2021; de Las Nv et al. 2023). Consequently, some international organizations have called for new policies, regulations, and updates to occupational health and safety guidelines to help prevent work-related stress (Hanson and Lang 2014). This evaluation study highlights the importance of the trainer’s expertise in the subject and the significance of presenting real-life cases. In both quantitative and qualitative evaluations, participants gave the highest scores to the trainer’s expertise and enthusiasm.

Recent research indicates that trauma-informed training positively influences professional relationships among healthcare workers. Studies have shown that such training enhances knowledge and ability to provide trauma-informed care, leading to greater satisfaction in professional relationships, especially in settings involving refugees (Fragkiadaki et al. 2020). Furthermore, it equips healthcare workers with effective strategies to support patients with traumatic experiences, thereby improving their professional relationships (Forber-Pratt et al. 2021). Training also significantly increases confidence in delivering culturally safe and responsive care, particularly to refugee and asylum seeker populations (Kelton et al. 2022). Additionally, trauma-informed training fosters a sense of community, empowerment, and healing, as it helps healthcare workers recognize and respond to trauma signs in both patients and colleagues (Elisseou 2023). Moreover, such training is associated with increased efficacy in trauma-informed care, especially among workers with higher levels of openness (Stevens et al. 2019). Lastly, it has been observed to improve organizational culture, enhance professional satisfaction, and foster greater empathy and camaraderie among colleagues (Damian et al. 2017).

The results of this study should be considered within its limitations. Firstly, using a valid and reliable measurement tool in the 6-month follow-up training evaluation would have been more advantageous. Nevertheless, no such tool with established validity and reliability was available in Türkiye at the time of the study. Therefore, an 18-item form based on the fundamental principles of trauma-informed care was prepared and used. Secondly, an important limitation of the study is the absence of a control group. Future studies are recommended to evaluate the differences between control and intervention groups. Thirdly, the study needs to be evaluated in the context of its proximity to a major earthquake event that serendipitously preceded the planned intervention study. The impact of the project may have therefore been enhanced by exposure of healthcare workers to extraneous information. Further research is required to develop appropriate measurement methods to assess the impact of trauma-informed training programs. In addition to service providers, it is recommended that new evaluation methods be developed to include patients, unit managers, and teammates where the trainee is based.

## Conclusion

Trauma-informed training helps healthcare workers understand the principles of trauma-informed professional practices, leading to a change in attitude, increased empathy and sensitivity, and the development of treatment plans with an interdisciplinary approach. Such an approach reduces stigma toward patients with traumatic experiences, enhances patient-centered care, and contributes to faster recovery, while also preventing occupational diseases and accidents among workers. Furthermore, the program’s demonstrated ability to significantly reduce burnout symptoms highlights the critical role such training plays in enhancing healthcare workers’ resilience, empowering professional practice, and improving interpersonal relationships. In-depth interviews indicate a need for more frequent and extended trauma-related training and a greater demand for experts in this field. Additionally, investigating the impact of training on practices and procedures and realizing organizational change are considered crucial steps. The results of this study also underscore the importance of adopting trauma-informed care initiatives more widely in disaster-affected regions, where they can enhance healthcare workers’ perceptions of service efficacy and value, ultimately transforming trauma-informed healthcare systems worldwide.

## Reflexivity statement

The authors are female. The authors are from Turkey. Each of the two authors lived and worked for >10 years in Turkey. Prof. Şimşek has been working at Istanbul Bilgi University as a manager, a lecturer, and a researcher on disasters and psychological trauma programs. Her major interesting area is to develop community-based prevention and recovery health management programs and evidence-based training materials based on human behavior change theories for vulnerable populations at the national level. The other author is a psychologist and working on trauma disasters.
